# Dose-Dependent Prebiotic Effect of Lactulose in a Computer-Controlled In Vitro Model of the Human Large Intestine

**DOI:** 10.3390/nu9070767

**Published:** 2017-07-18

**Authors:** Melanie K. Bothe, Annet J. H. Maathuis, Susann Bellmann, Jos M. B. M. van der Vossen, Dirk Berressem, Annalena Koehler, Susann Schwejda-Guettes, Barbara Gaigg, Angelika Kuchinka-Koch, John F. Stover

**Affiliations:** 1 Fresenius Kabi Deutschland GmbH, Else-Kroener-Strasse 1, 61352 Bad Homburg, Germany; 2 Triskelion B.V., A TNO Company, P.O. Box 844, 3700 AV Zeist, The Netherlands; annet.maathuis@triskelion.nl (A.J.H.M.); susann.bellmann@triskelion.nl (S.B.); 3 The Netherlands Organisation for Applied Scientific Research (TNO), Microbiology and Systems Biology Department, P.O. Box 360, 3700 AJ Zeist, The Netherlands; jos.vandervossen@tno.nl; 4 Fresenius Kabi Deutschland GmbH, Borkenberg 14, 61440 Oberursel, Germany; dirk.berressem@fresenius-kabi.com (D.B.); annalena.koehler@fresenius-kabi.com (A.K.); susann.schwejda-guettes@fresenius-kabi.com (S.S.-G.); john.stover@fresenius-kabi.com (J.F.S.); 5 Fresenius Kabi Austria GmbH, Estermannstrasse 17, 4020 Linz, Austria; barbara.gaigg@fresenius-kabi.com (B.G.); angelika.kuchinka-koch@fresenius-kabi.com (A.K.-K.)

**Keywords:** Lactulose, microbial fermentation, *Bifidobacteria*, lactobacilli, *Anaerostipes*, butyrate, ammonia

## Abstract

Lactulose, a disaccharide of galactose and fructose, used as a laxative or ammonia-lowering drug and as a functional food ingredient, enhances growth of *Bifidobacterium* and *Lactobacillus* at clinically relevant dosages. The prebiotic effect of subclinical dosages of Lactulose, however, remains to be elucidated. This study analyses changes in the microbiota and their metabolites after a 5 days Lactulose treatment using the TIM-2 system, a computer-controlled model of the proximal large intestine representing a complex, high density, metabolically active, anaerobic microbiota of human origin. Subclinical dosages of 2–5 g Lactulose were used. While 2 g Lactulose already increased the short-chain fatty acid levels of the intestinal content, 5 g Lactulose were required daily for 5 days in this study to exert the full beneficial prebiotic effect consisting of higher bacterial counts of *Bifidobacterium*, *Lactobacillus*, and *Anaerostipes,* a rise in acetate, butyrate and lactate, as well as a decrease in branched-chain fatty acids, pH (suggested by an increase in NaOH usage), and ammonia.

## 1. Introduction

Lactulose is a synthetic disaccharide consisting of galactose and fructose. It is indicated for the symptomatic treatment of constipation at doses of 10 to 30 g and the treatment of portal systemic encephalopathy at doses of 60 to 100 g. Furthermore, lower dosages than 10 g Lactulose are used as a functional food ingredient. Lactulose is neither digested nor absorbed from the upper gastrointestinal tract. Once it reaches the colon it is anaerobically fermented by the microbiota and serves as a prebiotic substrate by increasing the count of *Bifidobacterium*, *Lactobacillus*, and bacterial metabolites like short-chain fatty acids (SCFA) [1,2]. Hence, Lactulose can be characterized as a non-digestible carbohydrate with fiber-like effects. In healthy individuals, daily dosages of 10 g Lactulose already exerted beneficial effects on the human microbiota [3], as did even lower dosages like 4 g [4] and 3 g [5]. This raises the potential for the use of subclinical dosages of Lactulose, which do not have a strong laxative effect, exclusively for prebiotic effects. The minimal dose of Lactulose required for a prebiotic effect, however, remains to be determined. 

The above mentioned studies used different durations of treatments and different subject populations which limits the interpretation of dose-dependency of the prebiotic effect of Lactulose. Furthermore, direct effects of substances on the metabolic activity of the microbiota are difficult to determine in humans due to the substantial absorption and metabolism of products like short-chain fatty acids in the gut [6]. This drawback can be circumvented using the TIM-2 system, a computer-controlled model of the proximal large intestine representing a complex, high density, metabolically active, anaerobic microbiota of human origin. Thus, we aimed at the evaluation of the prebiotic effect of different dosages of Lactulose under controlled experimental conditions in the TIM-2 system.

In former studies with this system, daily 10 g Lactulose administration for 48 h led to a change in the SCFA ratio mainly based on an increase in acetate and a decrease in butyrate [2], which was most probably due to an increase in acetate producing *Bifidobacterium* and *Lactobacillus* species. When 7.5 g Lactulose were administered for 72 h to fecal samples of lean volunteers in this system, elevated levels of SCFA were observed in the Lactulose group as well and this dose of Lactulose mainly stimulated the growth of *Bifidobacterium* as well as *Alistipes* spp., *Parabacteroides* spp., *Parasutterella* spp., and *Anaerostipes* spp. [1]. No effect on *Lactobacillus* was reported in this group.

The current study is aimed at comparing daily dosages of 2 to 5 g Lactulose for 120 h in the TIM-2 system. In this study, we show that even 2 g Lactulose leads to an increase in SCFA, mainly reflected by acetate, while 3 or more grams of Lactulose are required to also observe an increase in butyrate. At 4 g Lactulose, the reduction in ammonia was as prominent as at a dose of 5 g, whereas an increase in relative abundance of *Bifidobacterium*, *Lactobacillus* and *Anaerostipes* was most evident in the 5 g Lactulose group. Our results show that a maximum daily dose of 5 g Lactulose for five days was associated with the full pattern of beneficial prebiotic effects on the intestinal microbiota.

## 2. Materials and Methods 

### 2.1. Test Product

The product administered in this study was Laevolac^®^ (Fresenius Kabi Austria GmbH, Linz, Austria), an oral solution containing 670 mg/mL lactulose. Experiments without the addition of Laevolac^®^ served as negative control.

### 2.2. Intestinal Conditions of the TIM-2 System

The TNO Intestinal Model (TIM-2) is a dynamic in vitro model of the proximal colon that has been previously published [1,2,7,8]. The TIM-2 system was inoculated with a dense and highly metabolically active colon microbiota of human origin. In the system the following standardized conditions were simulated: body temperature; pH in the lumen of the proximal colon (pH 5.8); anaerobiosis; delivery of a substrate from the ‘ileum’ (SIEM; Standardized Ileum Efflux Medium); mixing and transport of the intestinal contents; absorption of water and absorption of fermentation products, metabolites and other low molecular weight compounds (via a semipermeable membrane inside the colon model).

SIEM simulates material passing the ileocecal valve in humans, or in other words material reaching the colon. SIEM was prepared as described previously [2,7,8,9] and contained the major non-digestible carbohydrates (pectin, xylan, arabinogalactan, amylopectin, starch) found in a normal western diet as well as protein (bactopepton, casein), ox-bile, Tween 80 as well as vitamins and minerals. SIEM does not require pre-digestion and was added to the system at a speed of 2.5 mL/h. The speed of the dialysis liquid was 1.5 mL/min. During the experiment, the intestinal contents are mixed continuously by the peristaltic movements of the TIM-2 system. In order to simulate the transit of the chime from proximal to distal colon, 25 mL of the lumen is removed every 24 h and discarded.

Prior to the performance of each experiment the secretion fluids and dialysis solutions were prepared freshly, the pH electrodes calibrated, new membrane units installed and the system was inoculated (one day before the start of the test period) with a standardized microbiota of human origin. This standardized microbiota was prepared as described [7] using fecal donations from a group of 4 healthy volunteers (1 male, 3 females, age 38.8 ± 3.9 years; BMI (body mass index) 24.2 ± 1.5 kg/m^2^). Individuals provided signed informed consent prior to participation, were non-smokers and had not used antibiotics, prebiotics, probiotics or laxatives within 1 month before the donation. 

At the start of the adaptation period, the TIM-2 system was inoculated with approximately 30 mL of the standardized microbiota and 80 mL dialysis fluid. The microbiota was allowed to adapt to the model conditions and SIEM for 16 h. After the adaptation period the 120 h test period started, in which test product was added to TIM-2 in a daily dose.

### 2.3. Addition of Test Product

The test product was added to the system at daily doses of 2 g, 3 g, 4 g, and 5 g Lactulose, mixed in the SIEM, which was added throughout the entire test period. Each dose was studied in duplicate (*n* = 2), while the control experiment was carried out as quadruplicate (*n* = 4). The test period of the TIM-2 experiments lasted 120 h (5 consecutive days).

### 2.4. Sampling from TIM-2

Metabolites like the short-chain fatty acids, branched-chain fatty acids (BCFA), ammonia and lactate produced in TIM-2 were continuously removed from the lumen by a semipermeable membrane unit. This dialysate was collected at the start of the test period and after 24, 48, 72, 96, and 120 h. Volumes were measured and samples were taken from the dialysate.

Luminal samples taken at the beginning and end of the experiment (*t* = 0 h and *t* = 120 h) were used to investigate the composition of the microbiota. The samples were snap frozen in liquid nitrogen and stored at ≤−72 °C until analysis. 

### 2.5. Sodium Hydroxide Usage (pH)

The pH was kept at pH 5.8 by automatic titration with 2 M NaOH, the consumption of NaOH was monitored.

### 2.6. Short-Chain Fatty Acids and Branched-Chain Fatty Acids 

The dialysate and lumen fractions of TIM-2 were analyzed with gas chromatography for SCFA (acetate, propionate and butyrate) and BCFA (iso-butyric acid and iso-valeric acid).

For SCFA/BCFA, samples were prepared and analyzed as described previously [10]. Briefly, dialysate samples were directly used, lumen samples were centrifuged (12,000 rpm at 4 °C for 10 min). A mixture of formic acid (20%), methanol, and 2-ethyl butyric acid (internal standard, 2 mg/mL in methanol) was added to the supernatant. A 3 μL sample with a split ratio of 75.0 was injected on a GC-column (ZB-5HT inferno, ID 0.52 mm, film thickness 0.10 um; Zebron; Phenomenex, Utrecht, The Netherlands) in a Shimadzu GC-2014 gas chromatograph (Shimadzu Europe, Duisburg, Germany). 

### 2.7. Lactate and Ammonia

Samples for lactate and ammonia analysis were centrifuged as described above. In the clear supernatant, both l- and d-lactate were determined enzymatically (based on Boehringer, UV-method, Cat. No. 1112821035, Roche Diagnostics, West Sussex, UK). Ammonia was determined based on the Berthelot reaction [11] in which ammonia first reacts with alkaline phenol and then with sodium hypochlorite to form indophenol blue. In the currently used method, due to its toxicity, phenol was replaced with salicylic acid. 

### 2.8. 16S rDNA Amplicon Sequencing

The bacterial population in the TIM-2 samples was analyzed using Next Generation sequencing. Total DNA from the collected TIM-2 lumen samples at the start (*t* = 0 h) and at the end (*t* = 120 h) of the experiments was isolated as described [12] with some minor adjustments: The samples were initially mixed with 250 μL lysis buffer (Agowa, Berlin, Germany), 250 μL zirconium beads (0.1 mm), and 200 μL phenol, before being introduced to a Bead Beater (BioSpec Products, Bartlesville, OK, USA) for twice 2 min. To determine the recovery of bacterial DNA from the samples, a quantitative polymerase chain reaction (qPCR) using primers specific for the bacterial 16S rRNA gene was used. Changes in the microbiota composition were analyzed by using mass V4 16S rDNA amplicon sequencing. For 16S rDNA amplicon sequencing of the V4 hypervariable region, 100 pg of DNA was amplified as described [13] using 30 amplification cycles, applying F533/R806 primers [14]. Primers included Illumina adapters and a unique 8-nt sample index sequence key [13]. Amplicon yield, integrity and size was analyzed on a Fragment Analyzer (Advanced Analytical Technologies, Inc., Heidelberg, Germany). The amplicon libraries were pooled in equimolar amounts and purified using agarose gel electrophoresis and subsequent the QIAquick Gel Extraction Kit (QIAGEN, Hilden, Germany). Paired-end sequencing of amplicons was conducted on the Illumina MiSeq platform (Illumina, Eindhoven, The Netherlands).

Processing of the sequencing data was done using the Mothur pipeline. The differences between the two bacterial community profiles were identified by using the LEfSe (Linear Discriminant Analysis Effect Size) analysis [15]. The method is based on categorical non-parametric hypothesis test and Linear Discriminant Analysis (LDA) which is a mathematical technique to characterize the difference between classes. This is a method for metagenomic biomarker discovery and therefore it allows finding organisms that significantly can describe the differences between two microbial communities. For this a cut-off level of relative abundance of individual genera was included with 0.01% of total sequences. In the analysis, the different test conditions were each (as replicate) compared to the control experiments. This shows which genus became significantly more or less abundant as a consequence of a test product condition compared to the control.

### 2.9. Statistical Analysis

Due to the amount of experimental replicates (*n* = 2), no statistics were performed. Instead, mean values of the experiments were compared to mean values of the control experiments.

## 3. Results

### 3.1. Sodium Hydroxide Usage

During fermentation of carbohydrates the microbiota produces acidic metabolites (for example SCFA and lactate), therefore the use of NaOH during the experiments indicates the activity of the microbiota fermenting the SIEM plus the test product which were added to the TIM-2 system. 

**Figure 1:**
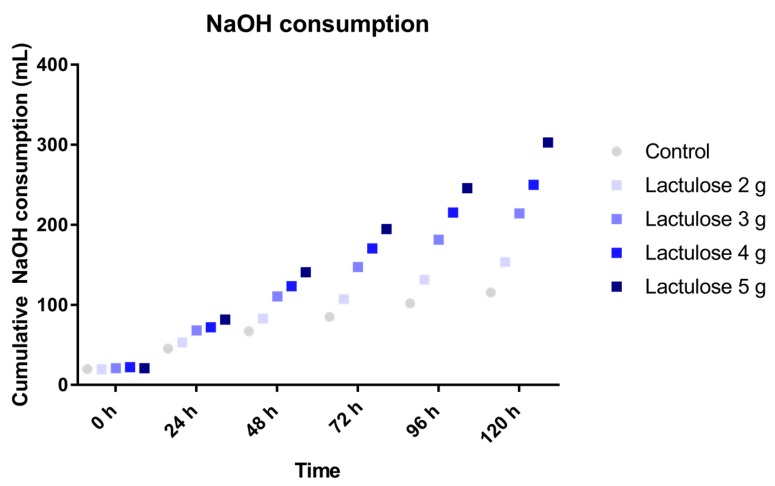
Sodium hydroxide cosumption during TIM-2 runs (mean of *n* = 2 (Lactulose dosages) or *n* = 4 (control)) with different dosages of Lactulose. Values at the start of the test period are on average 20.76 mL due to NaOH consumption during the pre-incubation period. All data points shown at the proximity of the individual time points indicated at the *X*-axis belong to these specific time points.

The addition of Lactulose in different doses in the test period showed an increased use of NaOH during the TIM-2 experiments as compared to the control as shown in Figure 1. The dose effect was clearly visible in the NaOH consumption as it was highest for the 5 g dose and decreasing per dose to the 2 g dose. The usage of NaOH was 303 ± 28 mL, 250 ± 19 mL, 214 ± 7 mL and 154 ± 9 mL for the experiments with 5 g, 4 g, 3 g and 2 g Lactulose, respectively.

### 3.2. SCFA Production

**Figure 2:**
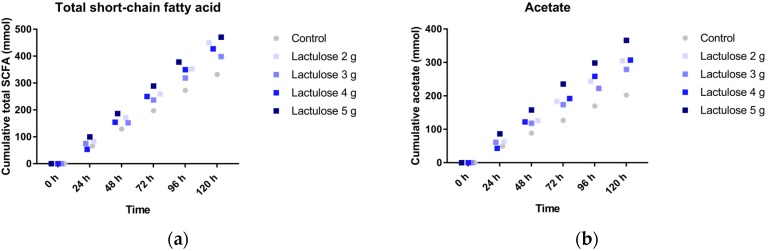

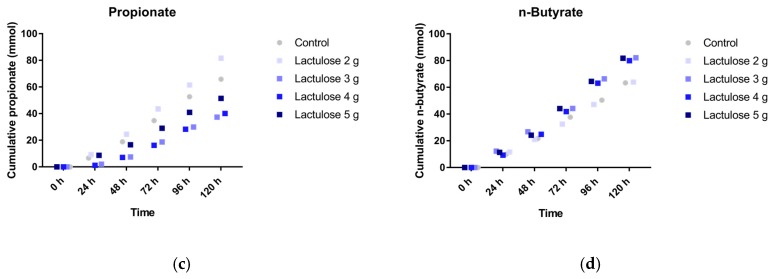
Production of (**a**) total short chain fatty acids (SCFA); (**b**) acetate; (**c**) propionate; and (**d**) butyrate in TIM-2 runs (mean of *n* = 2 (Lactulose dosages) or *n* = 4 (control)) with different dosages of Lactulose. Values at the start of the test period were set to zero. All data points shown at the proximity of the individual time points indicated at the *X*-axis belong to these specific time points.

Figure 2a shows the cumulative total SCFA (acetate, propionate and butyrate) production over time during the 120 h test period in TIM-2. The different Lactulose doses all show a higher SCFA production as compared to the control, with the highest SCFA production for the highest Lactulose dose of 5 g per day. The mean amounts of total SCFA produced for the increasing dose of Lactulose is 451 ± 3 mmol (2 g), 399 ± 21 mmol (3 g), 427 ± 76 mmol (4 g), and 471 ± 12 mmol (5 g), for the respective daily doses of Lactulose, respectively, compared to 332 ± 34 mmol (control).

The production profiles from each of the different SCFA measured (Figure 2b; acetate, Figure 2c; propionate and Figure 2d; butyrate), indicate that the acetate is the predominantly produced SCFA. The propionate production (Figure 2c) in the TIM-2 experiments with the higher doses (5, 4 and 3 g) of Lactulose was lower compared to the control. Whereas Lactulose tended to slightly increase butyrate production (Figure 2d) compared to the control as shown for the 3, 4 and 5 g dose (~80 mmol at *t* = 120 h). 

### 3.3. BCFA Production

**Figure 3:**
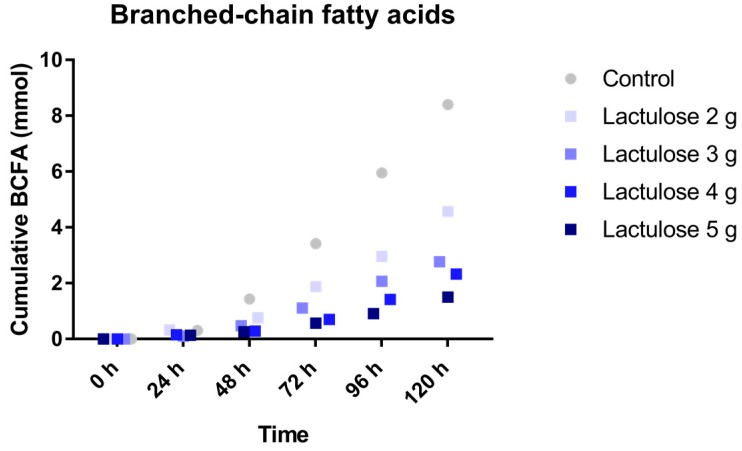
Cumulative branched-chain fatty acids (BCFA) (iso-butyrate and iso-valerate) production over time during the 120 h test period in TIM-2 runs (mean of *n* = 2 (Lactulose dosages) or *n* = 4 (control)). All data points shown at the proximity of the individual time points indicated at the *X*-axis belong to these specific time points.

The total production of BCFA in 120 h is shown in Figure 3. The BCFA production was reduced in the experiment when a higher amount of Lactulose was added to the TIM-2 system. BCFA production was 8.4 ± 4.2 mmol (control), 4.6 ± 2.2 mmol (2 g Lactulose), 2.8 ± 0.8 mmol (3 g Lactulose), 2.3 ± 0.7 mmol (4 g Lactulose), and 1.5 ± 0.2 mmol (5 g Lactulose).

### 3.4. Lactate

**Figure 4:**
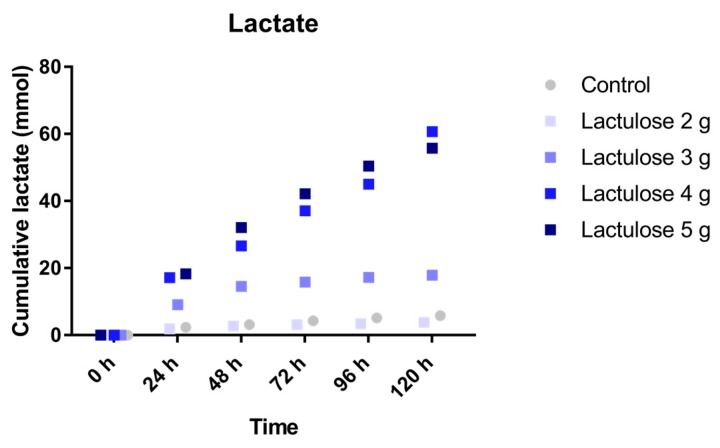
Cumulative lactate production over time during the 120 h test period in TIM-2 runs (mean of *n* = 2 (Lactulose dosages) or *n* = 4 (control)). All data points shown at the proximity of the individual time points indicated at the *X*-axis belong to these specific time points.

The cumulative amount of lactate (Figure 4) produced in the experiment with the lowest Lactulose dose (2 g per day, 3.8 ± 1.9 mmol) was similar to the control (5.8 ± 2.2 mmol). For the different doses of Lactulose, particularly at the highest dose, much higher amounts of lactate are formed during Lactulose fermentation. The amount of lactate produced appears to be dose-dependent. The highest lactate production was observed for the two highest Lactulose doses of 4 and 5 g per day. Lactate production was 17.9 ± 10.0 mmol (3 g), 60.7 ± 36.2 mmol (4 g), 55.7 ± 31.0 mmol (5 g) for the daily Lactulose dose, respectively.

### 3.5. Ammonia

**Figure 5:**
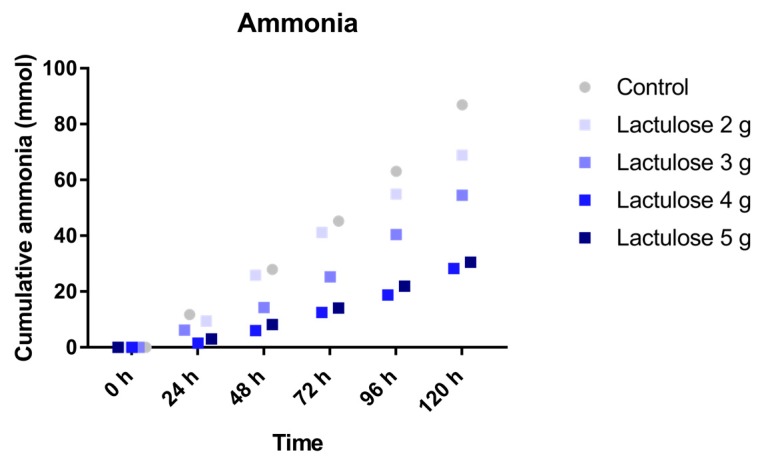
Cumulative ammonia production over time during the 120 h test period in TIM-2 runs (mean of *n* = 2 (Lactulose dosages) or *n* = 4 (control)). All data points shown at the proximity of the individual time points indicated at the *X*-axis belong to these specific time points.

The cumulative (total) amount of ammonia was measured for each of the test condition as shown in Figure 5. The mean ammonia production with the different Lactulose dosages decreased with rising doses of Lactulose. Ammonia production for the different test conditions was 87.0 ± 27.9 mmol (control), 68.8 ± 4.7 mmol (2 g Lactulose), 54.5 ± 5.8 mmol (3 g Lactulose), 28.3 ± 12.6 mmol (4 g Lactulose), and 30.5 ± 5.1 mmol (5 g Lactulose).

### 3.6. Microbiota Composition

Analysis with mass V4 16S rDNA amplicon sequencing resulted in an overview of the relative abundance of the different bacterial genera present in the microbiota lumen samples collected from the TIM-2 experiments after 120 h exposure to the different test conditions. The genus level abundance change for the control and test conditions after 120 h in TIM-2 is shown in Table 1. 

**Figure 6:**
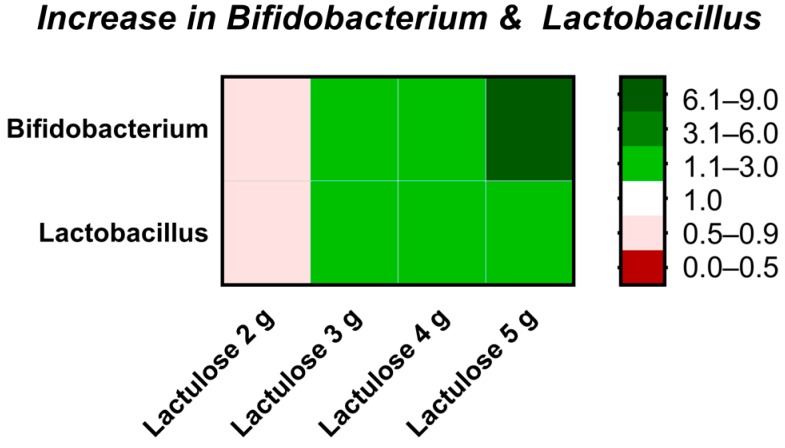
Heat map depicting fold changes of the increase in Bifidobacterium and Lactobacillus at different Lactulose doses compared to control after the 120 h test period in TIM-2 runs. A value equal to 1 (white) indicates no change, a value of >1 (green) indicates an increase, a value of <1 (red) indicates a decrease of the microbial genera.

**Figure 7:**
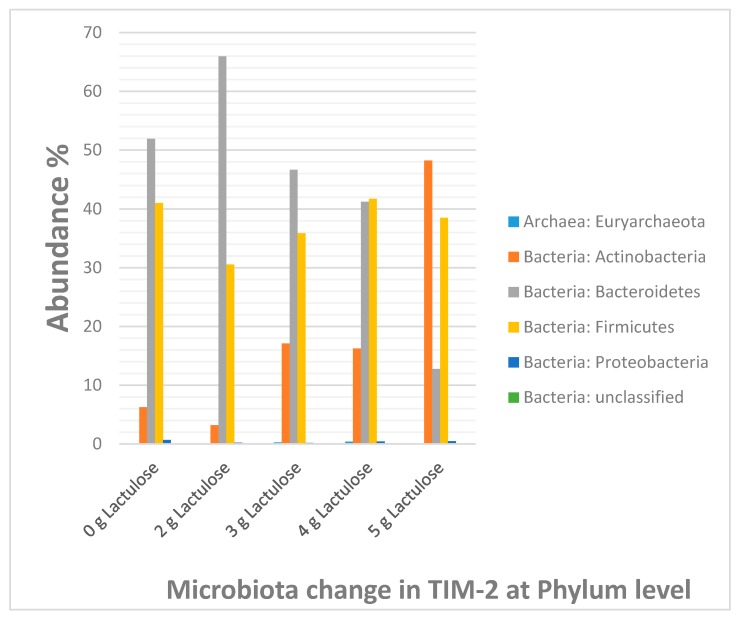
Column diagram of the bacterial distribution in percentage at the phylum level in control (0 g Lactulose) and different Lactulose dosages, 2 g, 3 g, 4 g and 5 g respectively. Apart from the 2 g lactulose data, an increase is observed with respect to the phylum *Actinobacteria* while a decrease is observed with respect to the phylum *Bacteroidetes*. The relative abundance of the *Firmicutes* is affected to a lesser extent.

The increase in the absolute bacterial count of the two genera Bifidobacterium and Lactobacillus is represented in form of a detailed heat map in Figure 6, in addition to the heat map for all analyzed species (Table 2) and the diagram for phylum shifting (Figure 7).

## 4. Discussion

This study showed that low dosages of Lactulose have an effect on the gut microbiota after 5 days of treatment in an in vitro model of the human proximal colon depicted by an increase in NaOH consumption suggesting a decrease in pH, decreased production of ammonia and BCFA as well as an increase in acetate, butyrate and lactate. With regard to dose-dependency, even dosages as low as 2 g Lactulose lead to a rise in SCFA, mainly acetate, and a correlating acidification of the intestinal content. At this dose, however, a slight decrease rather than an increase in the bifidobacteria or lactobacilli was observed with our methods compared to the control, indicating that the rise in SCFA is based on bacteria other than *Bifidobacterium* and *Lactobacillus* here. 

An increase in the relative amount of bifidobacteria and lactobacilli started at a dose of 3 g/day Lactulose. Moreover, at this dose, elevated levels of butyrate and a decrease in branched-chain fatty acids was observed after five days of treatment. Similar results have been described before, when healthy individuals treated with 3 g/day Lactulose for 2 weeks showed an enhanced growth of *Bifidobacterium*, but not *Lactobacillus* [5]. Neither short-chain or branched-chain fatty acids nor *Anaerostipes* were investigated in this trial [5].

When the TIM-2 system was inoculated with 4 g Lactulose per day, an additional increase in lactate and a marked decrease in ammonia levels were detected. When this dose was applied for three weeks to healthy humans, the increase in *Bifidobacterium* as observed with 3 g/day was again confirmed [4]. In addition, these volunteers showed a non-significant trend to higher levels of acetate, butyrate and lactate as well as a decrease in propionate and ammonia [4]. *Anaerostipes* was not analysed in this trial. 

In our study, the growth support of *Bifidobacterium* was most prominent at 5 g Lactulose per day, as was the increase in acetate levels, while the other effects like increase in butyrate were comparable to the 4 g dose. Bifidobacteria produce acetate and thus may be the basis for the high acetate levels observed in this dose group. A dose of 5 g Lactulose per day was administered for 10 days to healthy volunteers [4], however, neither the production of SCFA or ammonia nor the microbiota were analyzed in this dose groups. Therefore for the time being clinical trials administering 5 g Lactulose per day to healthy human volunteers are still missing for a comparison of these results to the in vivo situation. 

In the TIM-2 system, a dose of 10 g Lactulose led to lower butyrate levels after 48 h of treatment [2], suggesting a shift in bacteria to non-butyrate producing microbiota with accelerating dose of Lactulose. In healthy volunteers treated with 10 g/day Lactulose for 6 weeks, an increase in fecal *Bifidobacterium* was observed, while SCFA were not analyzed [3]. In another study, a higher dose of 20 g/day Lactulose for 4 weeks indeed led to decreased fecal butyrate levels [16]. 

These results indicate that 5 g/day Lactulose after 5 days of treatment leads to a balanced growth enhancement of *Bifidobacterium*, *Lactobacillus* and *Anaerostipes*, resulting in an increase in the metabolites acetate, butyrate and lactate as well as higher NaOH consumption, while ammonia and BCFA decrease. Although higher levels of butyrate and lactate as well as the decreased levels of ammonia are comparable to the 4 g/day dose, due to the most prominent increase in *Bifidobacterium* accompanied with a further increase in acetate the 5 g/day dose is considered superior to the lower Lactulose dosages. Higher dosages of Lactulose than 5 g/day bear the risk of decreased butyrate levels due to a one-sided increase in non-butyrate producing bacteria. 

The effect of high dosages of Lactulose on butyrate levels has been the subject of recent debate [1,2,16]. In our experimental setting 3, 4 and 5 g of Lactulose led to higher butyrate levels after 96 and 120 h. This is most probably due to an increase in butyrate producing *Anaerostipes* [17] at these dosages of Lactulose. Consistent with these high butyrate levels, lactate, which can be fermented to butyrate [18], was increased as well. In contrast, a decrease in butyrate was observed in another study with the TIM-2 system [2]. These studies investigated a higher dosage of Lactulose (10 g/day) for a different time period (7 days in vivo followed by 48 h in vitro or 48 h in vitro without in vivo pre-treatment). Interestingly, the butyrate levels of healthy volunteers treated for 7 days with 10 g/day Lactulose in this trial did not differ when analyzed directly after sampling, as did the samples from individuals without Lactulose pre-treatment after in vitro incubation with Lactulose for 48 h [2]. The fecal samples of the group pre-treated with Lactulose for 7 days, however, showed decreased levels of butyrate after 48 h in vitro incubation [2], indicating a dose and time dependency of this butyrate reducing effect of Lactulose. This is in line with the study of Ballongue and coworkers showing that 10 g/day of Lactulose lead to a decrease in butyrate levels in healthy individuals after 4 weeks [16]. In constipated patients, however, where this dose of Lactulose is mainly administered, no decrease in fecal butyrate levels was observed in patients treated for 4 weeks [19]. The reduction of butyrate levels seems therefore to occur mainly in healthy individuals, but not in constipated patients. In our study, focusing on evaluating the prebiotic effect of subclinical dosages of Lactulose, an increase and no decrease in butyrate levels was observed after 5 days of treatment, which is corroborated by a clinical trial investigating a low dose (4 g/day) of Lactulose for 3 weeks still leading to an increase in butyrate levels [4]. We therefore conclude that the administration of 5 g/day Lactulose is prebiotically beneficial in healthy individuals for up to 3 weeks, while a longer period of application will have to be evaluated in clinical trials in the future. 

In contrast to the butyrate levels the propionate production with Lactulose only showed an increase after addition of 2 g Lactulose, while 3 and 4 g Lactulose reduced the amount of propionate. A reduction, though not as extensive as with 3 and 4 g Lactulose, was also seen after addition of 5 g Lactulose. As for the butyrate levels, which may be based on the higher levels of *Anaerostipes*, this notion might be explained by the composition of the respective microbiota. For example, *Prevotella*, a propionate producing bacterium [20] with a relative abundance of more than 25% in our study, is slightly increased in abundance in the 2 g Lactulose groups, while the addition of 3 to 5 g Lactulose led to decreased levels of *Prevotella*. The slight increase in propionate in the 5 g Lactulose group compared to the 3 and 4 g Lactulose group might be accounted for by a strong increase in levels of other propionate producing bacteria in this dose group, like *Halomonas* [21], compensating for the propionate loss due to lower numbers of *Prevotella*.

Similarly, the increase in lactate, which is most prominent in the 4 and 5 g dose groups and surprisingly less extensive in the 5 g dose group compared to the 4 g dose group on day 5, can be explained by the composition of the microbiota. Lactate is the major product of lactic acid bacteria, including lactobacilli, bifidobacteria, enterococci, and streptococci [22]. Some of the lactate producing bacteria like lactobacilli slightly decreased in abundance in the 5 g group compared to the 4 g group and could account for the slight drop in lactate levels observed in this group at the fifth day of treatment. In addition some lactate-utilizing bacteria like *Shewanella* [23] or *Escherichia* [24] increased in the 5 g group and could contribute to this phenomenon.

BCFA are the product of protein degradation by proteolytic bacteria [25]. Relative abundance of such bacteria like *Bacteroides* is reduced after administration of Lactulose, a notion which might account for the lower levels of BCFA in the Lactulose groups.

In addition to healthy volunteers and constipated patients both benefiting from the prebiotic effect of Lactulose, this compound is also administered to patients with hepatic encephalopathy. The rationale for the treatment is the reduction of intestinal absorbable ammonia levels in order to decrease the total ammonia burden in these patients. Control of ammonia levels, is, however, also important for healthy individuals, as excess ammonia has been shown to change the morphology and metabolism of intestinal cells and reduce their lifespan [26]. In our study, the reduction of intestinal ammonia load was most prominent at dosages of 4 or 5 g Lactulose after 72, 96, and 120 h. In another experiment using the TIM-2 system, 7.5 g Lactulose for 3 days led to the lower ammonia levels (36 mmol) compared to apple fiber (65 mmol), sugar beet pectin (74 mmol) or galacto-oligosaccharides (44 mmol) in the stool of lean subjects [1]. These values are comparable to those observed in our study after 120 h with 4 or 5 g Lactulose, indicating that at least in the TIM-2 system an increase in Lactulose above 4 g/day does not lead to further improvement of the ammonia levels. In human healthy volunteers a daily dose of 3 g Lactulose for 14 days did not lead to significant improvement of total fecal ammonia [5]. This study, however, did not use the determination of a shift from urinary to fecal ^15^N-excretion, the current state of the art method [27]. Such a shift was observed in healthy individuals treated with a daily dose of 20 g Lactulose for 4 weeks [28], but lower dosages have not yet been investigated. Thus, the ammonia lowering effect of a lower dose of Lactulose like 4 or 5 g remains to be determined in a clinical trial. 

In conclusion, the results of this study clearly demonstrate a dose-dependent increasing positive effect of 2 to 5 g Lactulose on the intestinal flora and its metabolic activity. Starting with elevated levels of SCFA at low dosages (2 to 3 g), Lactulose further expands its beneficial impact on microbial composition and metabolism at higher dosages (4 up to 5 g). According to this experimental setting a dose of 5 g Lactulose per day is supposed to exert beneficial clinical effects like increase in *Bifidobacterium*, *Lactobacillus*, *Anaerostipes*, butyrate, acetate and lactate after 5 days of treatment. These results remain to be confirmed in a human study with healthy volunteers. 

## Figures and Tables

**Table 1:** Relative change of bacterial genera after 120 h fermentation experiments in TIM-2 (*n* = 2).

Genus	Relative Abundance (%)	Lactulose 2 g	Lactulose 3 g	Lactulose 4 g	Lactulose 5 g
**Growth enhancement**					
Bifidobacterium	19.8	0.5	2.62	2.37	7.96
Lactobacillus	6.7	0.86	1.86	2.84	1.98
Blautia	6.1	1.61	1.99	1.89	1.52
unclassified_Ruminococcaceae	1.6	0.54	1.88	1.23	1.11
Collinsella	0.8	0.71	5.26	7.41	3.11
Allisonella	0.3	0.66	1.38	1.46	1.06
unclassified_Clostridiales	0.3	0.74	1.01	1.27	1.14
unclassified_Erysipelotrichaceae	0.2	12	372	352	323
Clostridium_XI	0.1	0.8	1.2	1.6	2.4
unclassified_Bacteria	0.08	0	2	2	2
Methanobrevibacter	0.07	0.31	3.13	4.46	1.05
Ruminococcus2	0.06	1.36	8.36	14.73	6.18
Anaerostipes	0.03	>6	>5.5	>37	>39.5
Butyricococcus	0.02	0	2.8	2.2	9.8
Olsenella	0.01	4.86	6	11.43	7.71
unclassified_Coriobacteriaceae	0.01	1	6	3	9
**Growth reduction**					
Prevotella	25.9	1.35	0.95	0.84	0.25
Clostridium sensu stricto	3.9	0.14	0.33	0.62	2.05
Ruminococcus	2.7	0.18	0.11	0.03	0.03
Bacteroides	1.6	0	0.01	0.01	0.03
Weissella	1.3	0.99	0.38	0.24	1.4
Dialister	1.2	0.76	0.35	0.23	0.25
Acinetobacter	0.6	0	0	0	0
Escherichia/Shigella	0.5	0	0	0.55	2.18
Enterobacter	0.3	0.32	0.31	0.78	0.45
Peptoniphilus	0.3	0.19	0.15	0.3	0.55
Paraprevotella	0.3	0	0.09	0.1	0.4
unclassified_Enterobacteriaceae	0.2	0.58	0.12	0.62	0.16
Oscillobacter	0.2	0.12	0.05	0.04	0.21
Clostridium XIVa	0.2	0.14	0.22	0.71	0.63
Sutterella	0.2	0.09	0.32	0.53	0.28
Parabacteroides	0.1	0	0	0	0
unclassified_Prevotellaceae	0.1	0.26	0.58	0.76	0.64
Methanosphera	0.08	0.49	0.23	0.3	0.19
Succiniclasticum	0.06	0.06	0.02	0.13	0.03
Shewanella	0.06	0.15	0.31	0.92	6.62
unclassified_Clostridiales	0.05	0.03	0.23	0.03	0.56
unclassified_Firmicutes	0.05	0	0	0	0
Pseudomonas	0.04	0	0	0	0
Atopobium	0.02	0.05	0.18	0.36	0.32
Clostridium_IV	0.01	0.33	0	0	0
*Alistipes*	0.01	0	0	0	0
*Finegoldia*	0.01	0	0	0.27	2.53
**No clear dose-dependent effect**					
*Enterococcus*	14.0	0.27	0.7	1.53	4.51
*Faecalibacterium*	5.9	1.15	0.91	1.26	0.86
*Dorea*	0.9	0.41	0.94	1.24	0.61
unclassified_Lachnospiraceae	0.5	0.45	0.39	1.79	0.51
Staphylococcus	0.4	n/d	n/d	>0.5	n/d
Streptococcus	0.3	n/d	n/d	>0.5	>8
Moraxella	0.3	n/d	n/d	n/d	n/d
Anaerococcus	0.3	0.05	0.89	3.07	31.96
*Roseburia*	0.3	3.08	0.08	12.85	5.31
*Gemmiger*	0.3	0.13	0.39	2.08	0.18
Coprococcus	0.1	0.45	0.27	3.19	4.39
Halomonas	0.1	0.17	0.67	1.08	7
Corynebacterium	0.1	n/d	n/d	>0.5	n/d
Subdoligranulum	0.06	0.26	1.13	0.65	0
Lachnospira	0.06	1.38	0.03	0.74	1.69
Clostridium_XIVb	0.03	2	0	0.1	1.14
Catenibacterium	0.02	n/d	n/d	>2.5	>84
unclassified_Bacteroides	0.02	2	0	10	24
Sporobacter	0.01	n/d	n/d	n/d	n/d
Slackia	0.01	>0.5	n/d	>3	>2
Akkermansia	0.01	n/d	n/d	n/d	n/d

The ratio between the mean of the two runs of each Lactulose dosage and the control mean was calculated. Depicted are genera with a relative abundance of ≥0.01%. A value equal to 1 indicates no change, a value of >1 indicates an increase, a value of <1 indicates a decrease of the microbial genera. If the respective bacterial genus was not detected in control, but in the Lactulose groups, this is indicated with the prefix “>” in front of the value, whereas a value of zero states that the respective bacterium was below the detection limit in the Lactulose, but not in the control group. Bacterial genera below the detection limit in both the control and the Lactulose group are indicated with “n/d” for “not detected”. “Growth enhancement” and “growth reduction” refers to values > or <1, respectively, in at least 3 consecutive dosages of the 4 dose groups.

**Table 2:** The heatmap indicates the average relative number n of the different bacterial genera in the microbiota in the 5 different lactulose conditions 0 g, 2 g, 3 g, 4 g, and 5 g, respectively, after 120 h of exposure in TIM-2. The increasing reddish gradient color indicates the increase in the dominant genera in a particular sample (*n* > 1000), the yellowish gradient color indicates the genera that are present at an intermediate level (9 < *n* < 1000) while the increasing greenish gradient color represents the genera that are becoming more marginally present in the microbiota (*n* < 10).

Genus	Lactulose 0 g	Lactulose 2 g	Lactulose 3 g	Lactulose 4 g	Lactulose 5 g
Prevotella	12,069	16,305	11,493	10,138	3072
Bifidobacterium	1468	733	3842	3474	11,689
Enterococcus	272	75	191	416	1225
Lactobacillus	135	116	251	384	267
Blautia	1934	3112	3849	3654	2940
Faecalibacterium	1941	2227	1766	2448	1660
Clostridium_sensu_stricto	50	7	17	31	103
Ruminococcus	2535	457	267	80	65
Bacteroides	660	3	7	7	17
unclassified_Ruminococcaceae	581	315	1094	715	647
Weissella	40	40	15	10	56
Dialister	740	566	256	167	184
Dorea	415	169	390	515	251
Collinsella	70	50	368	519	218
Acinetobacter	1	0	0	0	0
unclassified_Lachnospiraceae	298	134	117	534	151
Escherichia/Shigella	3	0	0	2	6
Staphylococcus	0	0	0	1	0
Moraxella	0	0	0	0	0
Enterobacter	43	14	13	33	19
Anaerococcus	29	2	26	88	911
Peptoniphilus	178	35	28	54	99
Paraprevotella	43	0	4	5	17
Roseburia	7	20	1	84	35
Allisonella	53	35	73	77	56
Streptococcus	0	0	0	1	8
Gemmiger	162	21	63	338	29
unclassified_Clostridiales	128	95	129	163	147
unclassified_Enterobacteriaceae	63	36	8	39	10
Oscillibacter	286	34	15	11	60
Clostridium_XlVa	153	22	34	109	96
Sutterella	47	4	15	25	13
unclassified_Erysipelotrichaceae	1	6	186	176	162
Coprococcus	30	14	8	95	131
Parabacteroides	4	0	0	0	0
Halomonas	6	1	4	7	42
Corynebacterium	0	0	0	1	0
Clostridium_XI	1	1	2	2	3
unclassified_Prevotellaceae	69	18	41	53	44
Methanosphaera	13	7	3	4	3
unclassified_Bacteria	0	0	1	1	1
Methanobrevibacter	20	6	61	87	21
Ruminococcus2	6	8	46	81	34
Subdoligranulum	29	8	33	19	0
Lachnospira	20	27	1	15	33
Succiniclasticum	79	5	2	11	2
Shewanella	3	1	1	3	22
unclassified_Clostridia	15	1	4	1	9
unclassified_Firmicutes	3	0	0	0	0
Pseudomonas	0	0	0	0	0
Anaerostipes	0	6	6	37	40
Clostridium_XlVb	5	11	0	1	6
Butyricicoccus	3	0	7	6	25
Catenibacterium	0	0	0	3	84
unclassified_Bacteroidetes	0	1	0	3	6
Atopobium	11	1	2	4	4
Sporobacter	0	0	0	0	0
Clostridium_IV	2	1	0	0	0
Olsenella	2	9	11	20	14
unclassified_Coriobacteriaceae	1	1	3	2	5
Alistipes	0	0	0	0	0
Finegoldia	4	0	0	1	10
Slackia	0	1	0	3	2
Akkermansia	0	0	0	0	0
